# An ecological approach to understanding university English teachers’ professional agency in implementing formative assessment

**DOI:** 10.3389/fpsyg.2022.916980

**Published:** 2022-09-14

**Authors:** Yuhong Jiang, Jia Li, Qiang Wang

**Affiliations:** ^1^Foreign Languages College, Shanghai Normal University, Shanghai, China; ^2^College of Teacher Education, East China Normal University, Shanghai, China; ^3^School of Foreign Languages and Literature, Beijing Normal University, Beijing, China

**Keywords:** teachers’ professional agency, formative assessment activities, professional development, university English teachers, ecological approach

## Abstract

As a sub-realm of Language Teacher Psychology (LTP), teachers’ professional agency has gained significant attention from educational practitioners and teachers. The aim is to better discern teachers’ professional development and teaching effectiveness with a view to ensuring the quality of language teaching. International literature concerning teachers’ professional agency has noted a shift from knowledge training to vocational development in relation to teachers’ experience in decision making. Yet, little research so far has scrutinized this specific issue in Chinese university settings, and the real picture of teacher agency needs further exploration. Besides, the multidimensional and complex nature of agency identifies the overwhelming research work in understanding its contents in detail based on the previous perspectives from individuals, society, and time. To this end, the ecological understanding of professional agency reframed the theoretical basis of this study, prone to explore how teachers’ experience could be examined in relation to individual capacity, resources, and structural and contextual variables. The study was conducted in Chinese university settings in response to the research gap related to understanding professional agency. Quantitative and qualitative data from questionnaires, interviews, and classroom observations with 116 university teachers in China demonstrated that the enactment of professional agency rested on the interplay between temporal contexts, teacher capacity, and beliefs, especially in the instructional community. The findings revealed that university teachers at different stages of career development manifested variability in exercising their professional agency in relation to adapting or adopting existing teaching concepts, methods, or approaches. Mounting evidence revealed some enablers and constraints in relation to formative assessment, time impact, classroom interaction, and school culture. Emphasizing the interaction between individuals’ ability and their engagement with the professional environment, the findings provide insights into theoretical implications associated with ecological theory and enhance the practical discussion about promoting professional development for novice, mid-career, and veteran English teachers at the university level.

## Introduction

Language teachers in China often struggle with a multicultural teaching environment with unique educational and historical attributes (Littlewood, 2007; Butler, 2017). To address the educational gap, many countries have applied curriculum reform involving centralization and a top-down style (Wang and Cheng, 2005). In China, the requirements for higher quality education have accelerated the pace of further implementation of foreign language curriculum reform, making school education meet new demands and pedagogical challenges (Gao et al., 2018). For instance, the pandemic compelled university teachers to take up the technology challenge and adjust their teaching strategies for better student learning. The task of demonstrating improved language teaching competence has been applied to cultivate educators’ professional development, aiming not only to satisfy the requirements of being teachers but also to undertake the task of educational reform in classrooms. However, beginning and trainee teachers demonstrate different levels of agency, even in the same contexts, and language teachers, especially at their early career stage, often suffer professional burnout in the course of forging their teaching identity (Huang and Yip, 2021).

In addition to the language teachers’ professional development and pedagogical implications in response to teacher agency, shown as an ability to activate an individual’s inner mechanism to take stances which implies will, choice, freedom, and autonomy (Biesta and Tedder, 2007), little has been known about the picture of university teachers’ professional agency regarding how they make ecological decisions in language practices (Green and Pappa, 2021; Yang et al., 2021). The realm of foreign language teaching has succeeded in shifting significant attention toward professional agency, leading to a new phase of orthodoxy. Throughout the world, there is an urgent appeal in education for innovations that will allow university teachers to perform in an agentic, professional way. This has been raised as a strategic response to educational reforms and teaching environmental development (Eteläpelto et al., 2013). Therefore, a call has been raised for a special issue on agentic inspection of functional integration of emotions and decision making. In this regard, language teachers’ agency refers to teachers’ responses to plan and perform educational change, as well as to guide and conduct their teaching performance in educational contexts. To examine the variables related to teaching behavior and the educational environment, investigations into the agency associated with different periods of teachers’ careers have been conducted to unveil how agency contributes to the growing literature on teaching and learning (Pei and Yang, 2019; Kim et al., 2022) this can help to decide which method can be adapted for teachers to enhance their professional research and development. While some studies have been undertaken to discover the relatedness between teachers’ development on the one hand and school culture and leadership on the other (Postholm, 2012; Kovačević and Hallinger, 2019), there is a paucity of research on teachers’ experiences of being university teachers who agentively participate in formative assessment. Motivated by this gap, the present study aims to analyze the underlying structure of teacher agency in Chinese universities through the lens of an ecological approach and to explore how university teachers practice professional agency to meet the educational needs of formative assessment activities.

## Literature review

### Defining teacher agency

Agency has been defined from various perspectives. Fuchs (2001) delineated agency as individuals’ ability to perform actions in the context of sociocultural relatedness. Sewell (1992) and Hays (1994) conceptualized agency from the aspect of sociological influence, highlighting the significance of actors’ social functions (or choices) in contexts affected by the social structure. In this regard, individuals as members of society boast varying standards or qualities of agency, which can spark an infinite sense of potential in complicated social settings. Hence, the analysis of agency as the subtheme of psychology needs to include the privilege of scientific conceptualization, and a reexamination involving individuals’ interactions with peers, society, and family could provide a clear reflection on these individuals’ emotional development.

The history of agency research can be traced to social science (Emirbayer and Mische, 1998; Priestley et al., 2015, 2016) and anthropology (Ahearn, 2001; Holland et al., 2003); some psychology research (Bandura, 2001, 2006; Silbereisen et al., 2007) has also mentioned the possibility of an active and agentic role of action in educational practices. In an anthropological setting, Ahearn (2001) criticized the common definition of agency as free will (ignorance of social impacts on the nature of agency and pervasively influent cultural factors on human actions, beliefs, and reactions) and identified agency as resistance (feminism’s tendency to highlight the restraining power of gender inequality while underrating the resisting ability in individual cases). In light of other disciplines, Ahearn (2001) stressed the importance of linguistic interaction with the implication of practice theory, which treats human agency and structure as mutually constitutive. In a psychological study, Bandura (2006) adopted social cognitive theory from the point of view of the agentive role in human development and progress. From a psychological perspective, individuals are not just products of the environment but also contributors to their surroundings. Based on psychological theory, four core properties of human agency have been categorized these are *intentionality, forethought, self-reactiveness*, and *self-reflectiveness*.

In the realm of social science, the debate about agency has maintained its practical, albeit vague, significance that emphasizes the need to interpret the interpenetration between agency and structure. To this end, some scholars have suggested close contact with the voluntarism of rational choice (Perrotta, 2017), aiming to conceptualize agency’s different dimensions and its manifestations of the social structure in various ways in the sense that individual agency shapes social action (Lockton and Fargason, 2019; Sang et al., 2022). Different conceptualizations, such as Giddens’s (1984) “structuration” and Bourdieu’s “habitus,” have gradually formed the relatedness and mutual integration between individuals and their social surroundings. Giddens intended to make a resolution to put forward the nature of structuration, whereas Bourdieu endeavored to understand the ways in which individual preferences, actions, and behaviors played mutually vital roles in reproducing and underpinning social structures and individualism. These attempts have been criticized for misinterpreting the power of free will (Ahearn, 2001) and separating individual elements from macro-social reality (Layder, 1997). They both fail to exploit social development and transformation (Sewell, 1992), a discussion that is more amenable to social psychology. Throughout the history of sociology, the “agency and structure” debate guided a viable solution to tackle the problem of person and society, but it failed to take social psychological factors into account (Hitlin and Elder, 2007). Psychologically speaking, individuals are active social contributors, not merely products of life circumstances (Bandura, 2001). In this regard, social cognitive theory abandoned and condemned the concept of the duality of agency and social structure (Bandura, 2006). Owing to their advanced abstract cognitive capacity for symbolizing, individuals have developed into active agentic species with characteristics of self-governance, self-restraint, and proactiveness, enabling the presence of construction and evaluation of their environmental circumstances. Apart from four essential segments of agency in the light of social cognitive theory (*intentionality, forethought, self-reactiveness, and self-reflectiveness*), three modes of agency are intriguing and worth deep exploration; these are the individual, proxy (mediated), and collective modes of agency.

Other voices and conceptions of the nature of agency have also emerged (Lipponen and Kumpulainen, 2011; Hökkä et al., 2012). Recently, the sociocultural approach to understanding the individual and collaborative sense of creativity has been signified by scholars who have struggled with subjective development (Eteläpelto et al., 2013; Connolly et al., 2018). Activity theory, proposed by Leontiev (2020) with object-oriented approaches, introduces three levels of human activities, denying the agentic potential of human agency this treatment renders the existence and nature of agency ever more complex. The alternative subject-oriented developmental theory later becomes more appropriate to address the role of individuals and its relatedness to the changefulness and complexity of social and cultural contexts. This object-to-subject alternation calls attention to personal autonomous actions and their consequences for the individual’s surroundings, clarifying the importance of intentionality and individual subjectivity. Billett (2006) also jumped on the overemphasis of situational objective factors and recommended agency as an essential role in life-course learning and cultural transformation. In the theories of human learning and empirical investigation in social practices, the need to consider an individual’s intentions, preferences, identities, and interests stands out, meaning that participants’ agentic role should enjoy central and essential priority in a social environment. Put differently, the individual’s professional identity affects the efficiency of the agency, and the agency in turn plays a role in reconstituting and reshaping identity (Vähäsantanen and Eteläpelto, 2011; Teng, 2019).

### An ecological perspective on teachers’ professional agency

In terms of some implications and interpretations of professional agency, it is assumed that the way participants act, develop, and learn is constrained by socio-cultural contexts. There is a reciprocal, constructive relationship between the social context and an individual’s construction of behaviors and learning. The analytical process of agency can be realized with individuals’ interpretations and actions with regard to the specific environment in which agency is not isolated but is still the element that varies with social conditions. More specifically, an ecological approach to understanding agency manifests “an individual’s situated capacity in the course of decision making and acting with the interplay between personal capacity and the resources, affordance, and constraints of the environment” (Priestley et al., 2015). Priestley et al. (2015, 2016) analytically separated agency into some component dimensions and integrated its meanings with social engagement from the past (in terms of “*Iteration*”), the present (seen as the “*Practical-Evaluative*” competence to contextualize past manners and future perspectives within the current social settings), and the future (viewed as the “*Projective*” ability to foresee the alternative choices within the context). Hence, in light of the ecological perspective, it is suggested that agency needs to be critically and scientifically investigated within the interrelatedness of an individual’s capacity and professional environment in which personal achievement and development are relevant to this agentic ability.

Professional agency, viewed as embracing the power to affect, act, and initiate actions by making decisions, is germane to teachers’ professional occupation (Eteläpelto et al., 2013). In addition, how individuals enact and exert agency can be generalized as a dynamic procedure integrated with varying restraints or complexities of social contexts (Lipponen and Kumpulainen, 2011). This conceptualization has brought new ideas and insights for the context-driven analysis of professional agency. In ecological conditions, teachers’ professional agency is defined as teachers’ capacity to react to the constraints and affordances they encounter with pedagogical beliefs and definite purposes that render the desired positive outcomes in educational contexts (Davies, 1990). Teachers enact and exert professional agency by means of the interplay between pedagogical situations and individual capacity when they respond to educational changes, involving either affordances or constraints in decision making (Tao and Gao, 2021; Jeon et al., 2022). Pedagogical resources (i.e., affordances) in the teaching environment include educational support from peers or the school, teaching tools, technology use, educational policy, students’ features, or academic development along with teaching guidance; in contrast, constraints related to teaching practices adopted in response to pedagogical challenges (White, 2018; Jeon et al., 2022). Therefore, with respect to pedagogical challenges in university contexts, teacher agency is enacted in the specific contexts in the present time (practical-evaluative dimension), affected by personal and professional knowledge (iteration dimension) based on life history and their future aspiration (projective dimension). In addition, teachers in supportive or competitive communities can evaluate and reflect on their educational practices and take a practical stance for the sake of altering pedagogical situations and better academic achievement, which can either be “enabled or constrained” (Priestley et al., 2016; Huang and Yip, 2021; Jeon et al., 2022). In this regard, this study employed Priestley et al. (2015)’s agency model as the basis for analyzing and investigating ecological implications in response to pedagogical challenges arising in the context of university teaching.

### Teachers’ professional agency in formative assessment activities

Formative assessment was designed to develop students’ learning (Wu and Jessop, 2018), and both students and teachers can promise all activities in which informative feedback was provided to amend and improve teaching for satisfying students’ needs (Black and Wiliam, 2010). Formative assessment is effective in improving university students’ performance (Fuller, 2017; López-Pastor and Sicilia-Camacho, 2017). For freshmen in college, formative assessment’ beneficial role in their academic development has called for ample attention to the integration of feedback information and course instruction in university (McCallum and Milner, 2021). Further, Khajeloo et al. (2022) displayed the challenges and realizations of practicing the formative assessment within the university biological context, which was conducted through classroom observation, interviewer, and student focus group. The investigation unveiled that individuals’ practical assessment theories had implicational effects on formative assessment. It still conveyed differences in teachers’ responses based on the teaching context elements. In the light of the effectiveness of formative assessment in teaching experience, Verberg et al. (2016) examined the potential of teacher agency in the context of formative assessment with in-depth analysis, exploring how teachers in the formative assessment process formed a sense of agency in a teaching context. Their experiment focused on the relationship with the agency and whether teachers took an active role in the assessment process. Teachers had a strong sense of professional agency in assessment, albeit in such a way that the enactment of its agentic mechanism did not receive the uninterrupted privilege along with the whole teaching practices.

## Research design

### Research gap and questions

In light of the previous review and theoretical investigation of the nature of teacher agency, this study significantly explored how teachers’ professional agency supported the practice of formative assessment activities guided by the ecological perspectives in the university English classroom. The specifically detailed research questions are as follows:

*RQ 1: How do university English teachers exert their agency within the context of the formative assessment in the English teaching classroom?*

*RQ 2: What factors mediate the realization of teachers’ professional agency within the context of the formative assessment in the* university *English teaching context?*

### Research participants and research site

For the sake of more accurate inquiry into the agentic impacts on university English teachers’ professional decision-making, this study conducted an empirical experiment in the context of Chinese universities with different teaching experiences, gender, educational background, and professional titles, among others. The study did not focalize one target university as the same working environment might affect and confine the diversity of teachers’ responses in the research. Hence, the participants could have totally different organizational support and the research context could enjoy the multiformity, complexity, and confluence of teachers’ working environment, for the professional agency need to be comprehended from the perspective of a structural dimension which includes relationships, roles, and trust among their teaching environment. In accordance with the research content, the research focused on the university teachers and testified how they made their decisions in response to different teaching contexts. The quantitative research sample encompassed 116 university teachers with 22 male teachers (18.97%) and 94 female teachers (81.03%) from different provinces and municipalities (for instance, Shanghai 65/56.03%, Gansu 21/18.10%, Yunnan 8/6.90%, Jiangsu 5/4.31%, Beijing 3/2.59%, et al.). In addition, ten English teachers were chosen for the qualitative study (Table 1) based on their different basic information (mostly gender, educational background, and teaching years), aiming to attain an in-depth understanding of professional agency.

**TABLE 1:** English teachers’ basic information in qualitative research.

Name	Code	Gender	Educational degree	Teaching year
Interviewee 1	IN1	Female	Master’s degree	26
Interviewee 2	IN2	Female	Ph.D.	6
Interviewee 3	IN3	Male	Ph.D.	5
Interviewee 4	IN4	Female	Master’s degree	19
Interviewee 5	IN5	Female	Master’s degree	20
Interviewee 6	IN6	Male	Ph.D.	6
Interviewee 7	IN7	Female	Master’s degree	10
Interviewee 8	IN8	Male	Ph.D.	8
Interviewee 9	IN9	Female	Master’s degree	24
Interviewee 10	IN10	Male	Ph.D.	2

### Research instruments

#### Questionnaire

In light of the previous ecological review of agency, the enactment or achievement of agency can be described as being affected by multiple components, such as individual affections, resources, and cultural and structural influences (Leijen et al., 2021). Given the ecological model (Priestley et al., 2015) of agency, which encompasses three different subcategories-iterational, projective, and practical-evaluative-the questionnaire was created and developed to elucidate teachers’ agency in three domains, that is, planning of teaching and learning activities, teaching students of diverse ability in the same class, and using formative assessment activities in teaching. These domains were chosen because agency is a domain-specific and activity-targeted element in many significant aspects of teaching work. Planning teaching and learning activities is vital for the healthy development of the teaching process in classroom design and needs to be carried out in a prepared manner. Using formative assessment for teaching students of diverse ability meets the sharply increasing demands for teachers, especially in university English teaching contents.

In a quantitative approach, the design and structure of the questionnaire were based on the ecological model as the theoretical framework, in line with the important characteristics of university English teachers’ development in their professional teaching practices. The whole survey consisted of two components-namely, questions on demographic characteristics and a survey on university English teachers’ professional agency. The first part investigated the targeted university English teachers’ personal information, including gender, age, years of teaching, personal educational background (received university degree), and other aspects. The second part comprised multiple choice questions from three different dimensions, adapted from the Teachers’ Professional Agency Survey with an ecological focus (Leijen et al., 2021). The whole survey consisted of 30 statements from the three teachers’ practice-related domains, and each component was divided into three different angles based on the ecological model; these angles were the iterational (past experience), projective (futural prospect), and practical-evaluative dimensions (cultural, material, and structural conditions of the current situation). The teachers targeted in the survey were asked to rate the extent to which they agreed or disagreed with the statement on a five-point Likert-type scale, ranging from 1 (strongly disagree) to 5 (strongly agree). The reliability coefficient value was 0.934; since this value is higher than 0.9, it indicates that the reliability of the research data was of high quality. Apart from the reliability analysis of the questionnaire, the KMO and Bartlett’s test results (=0.878 and spheroidal consequence = significant) demonstrated the high credibility of the questionnaire (as in Supplementary material).

#### Semi-structured interview protocol

The in-depth face-to-face interviews about the perception of professional teacher agency were conducted to unpack the nature of psychological activities and choices based on the teaching experience and futural expectations. Also, this interview is designed to refine, assist, and perfect the discovery of quantitative research, for it enjoys strong flexibility and can obtain direct and reliable data and materials without language and time limitations. The whole part is divided into five main sections, and each component challenges several central problems or statements.

### Data collection and data analysis

To ensure validity, each component in the questionnaire was scientifically examined and worded for the purpose of measuring the real meaning of teacher agency in professional development. The research administrated and distributed the questionnaire to the targeted teachers at the initial time of the semester, for it enabled them to plan and assess the formative activities in the teaching practices and to promptly survey some basic background information about their age, education, and degree.

After the preparation work of the questionnaire, the experimental raw data were carefully examined and coded to exclude some useless retrieved data. The quantitative data analysis was principally conducted with the Statistical Package for Social Science (SPSS). This analytical software has been praised for its automatic statistical mapping, in-depth data analysis, and complete functions. Based on the questionnaire guide of agency-targeted questions, the independent sample *t*-test and one-way ANOVA were utilized to describe the disparity between teachers’ current situation and their professional teacher agency in teaching to discover the essence of agency in formative assessment activities.

**FIGURE 1:**
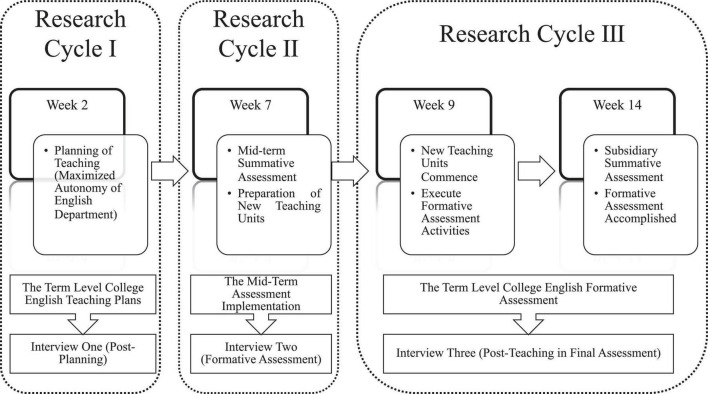
Research cycles of qualitative research.

As part of the research cycles, semi-structured interviews and observation context analysis were conducted throughout the school term three times with teacher volunteers. The structure of research cycles comprised interviews with subjects and observations of the classroom context. Poulton (2019) also utilized a research cycle to conduct a case study in a large metropolitan school in Australia to unveil the experiences of teacher agency in the top–down, bottom–up curriculum reform. The completed survey of research cycles provides new perspectives through interviews, for it enables to obtain interviewees’ mental activities, their interactions, and their behavioral results within the given context. Moreover, the interactive participation in the case study gives the researcher more opportunity to comprehend the inner complex essence of teachers’ moral engagement and conflicts (Cohen et al., 2007). In this university teacher investigation, the design of the interview involved three research cycles, which detail teachers’ planning of teaching activities, engaging in teaching innovation, and enacting their professional agency in decision-making as seen in Figure 1. The details in the figure could unveil how three research cycles are implemented within the reach of the research purpose.

Initially, the first 2 weeks function as “facilitators” of planning activities, aiming to maximize teachers’ autonomy in curriculum design and teaching planning. Being authorized to make some modifications to teaching materials, considerably or slightly, university teachers have more alternatives in classroom teaching, hence the necessity of planning analysis in week two stands out for classroom effectiveness. In university teaching settings, teachers tend to grade students’ achievement with the combined assessment methods (normally formative and summative), and more and more educators have addressed teachers’ vital and constructive role during different phases of teaching implementation and knowledge construction. Accordingly, some qualitative data could be retrieved from midterm and final term during the 2021 school year in such a way that post-planning and post-teaching activities could be blended together to unveil the essence of professional agency.

### Research road

**FIGURE 2:**
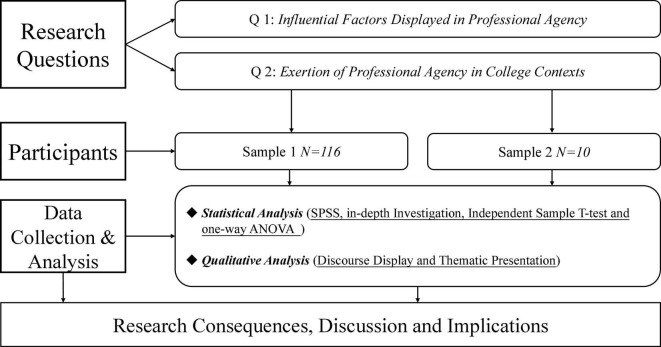
Research road.

Figure 2 has substantially introduced the analytical paradigm of the research, stressing the importance of the mixed methods to interpret the nature of the teacher agency way more than what can be observed simply from the previous illustration. With the employment of the designed plan, the productive use of quantitative and qualitative case studies in justification was outlined, in which teachers were free and motivated to participate in the research. The study highlighted the formative assessment activities in the English teaching class and how teacher agency was interplayed with the application of activities in the university context. The guide to conducting quantitative and qualitative research was introduced within the research circle as well.

## Research results

### Results of general level of university English teachers’ agency

In relation to the ecological perspective, the design of the questionnaire was split into three components, including planning, practicing, and assessing; this enabled the main characteristics in the process of formative assessment activities to be captured and insights from the implementation of formative assessment during course designing, practicing, and evaluating processes to be acquired. The planning section refers to the process in which teachers share the success criteria before carrying out the teaching practice and diagnose the students’ current level based on student data. In the classroom, teachers adopt various strategies to assure the effectiveness of the classroom, including classroom questioning, advocating peer and self-assessment, and marking the improvement only by comments, among other things, as described in the section of practicing. The last part mentioned the method used to assess the students’ performance and explored the extent to which teachers apply the strategy of formative use of summative tests.

Prior to displaying the overview consequences from the collected data, the statistics were drawn into analytical software to unveil the potential relations between variables in teaching. Afterward, the descriptive statistics of teacher agency in formative assessment are presented in Table 2.

**TABLE 2:** Descriptive statistics of agency in the different processes of formative assessment.

Process of formative assessment activities in university English classroom	Min	Max	Mean	*SD*
***Agency in Planning of Teaching and Learning Activities (Sharing Success Criterion with Students)***	***2.10***	***5.00***	***3.91***	***0.81***
Variable 1: Iterational Dimension	1.00	5.00	4.20	0.69
Variable 2: Projective Dimension	1.00	5.00	4.23	0.71
Variable 3: practical-Evaluative Dimension	1.00	5.00	3.45	0.99
***Agency in Practicing Classroom Activities (Interaction, Feedback)***	***1.70***	***5.00***	***3.67***	***0.87***
Variable 1: Iterational Dimension	1.00	5.00	3.97	0.80
Variable 2: Projective Dimension	1.00	5.00	3.81	0.77
Variable 3: Practical-Evaluative Dimension	1.25	5.00	3.34	1.00
***Agency in Formative Use of Summative Tests***	***2.20***	***5.00***	***4.03***	***0.77***
Variable 1: Iterational Dimension	2.67	5.00	4.30	0.70
Variable 2: Projective Dimension	3.00	5.00	4.19	0.68
Variable 3: Practical-Evaluative Dimension	1.25	5.00	3.72	0.89

Bold values indicate different domains and variables of agency in formative assessment.

Hinged upon the statistic results from database, it shows that university English teachers have the relatively highest level of agency in the activities of formative use of summative tests (*M* = 4.03, *SD* = 0.77), the relatively lowest agency in practicing classroom activities (*M* = 3.67, *SD* = 0.87), and a medium level of professional agency in planning teaching and learning activities (*M* = 3.91, *SD* = 0.81). As seen in Table 2, three subthemes (planning, practicing, and assessing) have different demonstrations, but the practical-evaluative dimension (cultural, structural, and material variables) contributed the lowest in the enactment of teachers’ professional agency as *M* = 3.45 in planning, *M* = 3.34 in practicing classroom activities, and *M* = 3.72 in formative use of summative tests.

### Results of professional agency status of university teachers based on various categories of demographics

This section aims to unveil the relatedness of demographic variables and the enactment of professional agency in different periods. To this end, independent *t*-tests (i.e., gender) and ANOVAs (i.e., major) were conducted. Demographic characteristics refer to the participants’ basic personal information about gender difference, age disparity, professional title, and educational qualifications. The research data were retrieved and imported into SPSS for statistical analysis.

#### Results of relation between teacher seniority and professional agency

An analysis of variance was conducted to describe the differences between variables of teaching experiences in exercising professional agency. The results demonstrated that there was no significant difference with no linear trajectory in planning practices. Similarly, the other two teaching activities, practicing and formative assessment in the final semester, did not witness any significant importance in iterational, projective, and practical-evaluative dimensions, indicating that teaching experience would not affect teachers’ enactment of professional agency in those two activities significantly.

#### Results of gender impact on teacher agency

The *t*-tests were applied to take stock of the relatedness between sexuality and the enactment of professional agency in planning, teaching, and assessing activities. Table 3 identified different professional agency scores of university English teachers of different genders. As seen in the following table, there are no significant differences in professional agency scores among university teachers of different genders. However, the details of statistics could illustrate the (stable or erratic) fluctuation of enactment of professional agency from curriculum designing and teaching activities planning to teaching effectiveness assessment. Female university teachers enjoyed the stable rising trajectory (*M* = 4.22, *SD* = 0.71) in planning, (*M* = 4.24, *SD* = 0.8) in practicing, and (*M* = 4.27, *SD* = 0.68) in assessing, but male teachers demonstrated the mixed and disorderly state of enactment of professional agency. Furthermore, female teachers more regular exercise their agentic choices in teaching activities, as the likely picture in three dimensions. Male teachers do not have the common changing trend, but female teachers have the strongest agency in the iterational dimension, a medium level agency in the projective dimension, and the lowest agency in the practical-evaluative dimension with a descending trajectory.

**TABLE 3:** Gender differences in the enactment of agency.

Items	(Mean ± SD)		*p* *
	Male (*n* = 22)	Female (*n* = 94)	
**Planning Teaching and Learning Activities**	**4.20 ± 0.82**	**4.22 ± 0.71**	**0.59**
*Iterational Dimension*	4.22 **±** 0.75	4.33 **±** 0.75	0.66
*Projective Dimension*	4.22 **±** 0.77	4.29 **±** 0.66	0.41
*practical-Evaluative Dimension*	4.17 **±** 0.91	4.17 **±** 0.72	0.68
**Implementing Teaching and Learning Activities**	**4.16 ± 0.65**	**4.24 ± 0.80**	**0.61**
*Iterational Dimension*	4.38 ± 0.59	4.33 ± 0.71	0.63
*Projective Dimension*	3.95 ± 0.92	4.28 ± 0.68	0.78
*practical-Evaluative Dimension*	4.14 ± 0.50	4.13 ± 0.96	0.47
**Using Formative Assessment in Teaching and Learning Activities**	**4.27 ± 0.69**	**4.27 ± 0.68**	**0.69**
*Iterational Dimension*	4.28 ± 0.69	4.43 ± 0.53	0.61
*Projective Dimension*	4.20 ± 0.79	4.33 ± 0.72	0.62
*Practical-Evaluative Dimension*	4.21 ± 0.75	4.21 ± 0.71	0.79

*p < 0.05 and **p < 0.01. Bold values indicate different domains and variables of agency.

#### Results of age and teachers’ professional agency

Table 4 depicts the variance of enactment of professional agency in terms of planning, practicing, and formative use. There was a significant difference in teaching practice and guidance in classroom settings, as addressed in the iterational dimension (*P* = 0.006*<0.05). The table indicates that age could impact the enactment of professional agency in teaching, since years of teaching undergirded teachers’ professional histories and teachers who desired to get professional promotion enacted strong professional agency in implementing curriculum design and classroom teaching for approaching the publication threshold. However, in the planning and assessing section, there were no significant differences, which pinpoints that years of teaching would not influence teachers’ professional agency when in planning and assessing, probably because, to some extent, these two activities were much more mechanical compared with classroom teaching activity.

**TABLE 4:** The difference in demographic variables in teacher agency.

Age	25–30 (*n* = 2)	30–35 (*n* = 16)	36–40 (*n* = 35)	Over 41 (*n* = 63)	p*
*Practicing: Iterational Dimension*	2.50 **±** 0.71	3.31 **±** 0.95	3.97 **±** 0.75	3.73 **±** 0.83	0.006*

**Professional title**	**Assistant (*n* = 3)**	**Lecturer (*n* = 67)**	**Associate professor (*n* = 42)**	**Professor (*n* = 4)**	***p*** *

*Planning: Iterational Dimension*	4.33 ± 0.91	4.06 ± 0.78	3.86 ± 0.89	4.58 ± 0.83	0.047*
*Planning: Projective Dimension*	4.34 ± 0.77	4.10 ± 0.80	3.73 ± 0.95	4.25 ± 0.93	0.011*

*p < 0.05 and **p < 0.01.

Considering the small number in groups 25–30 (*n* = 2), the discussion of teachers’ professional agency varying with age difference excluded the first crowd and concentrated on the other groups. Broadly speaking, university teachers (36–40 years old) exercised the strongest professional agency in planning, practicing, and formative use of summative assessment in terms of iterational, projective, and practical-evaluative dimensions.

#### Results of professional title effect on teacher agency

ANOVA was employed to testify to the interrelatedness between teachers’ professional titles and the enactment of professional agency in terms of different activities. Table 4 illustrates the comparative trajectory of how teachers exercised their professional agency in language teaching. From the statistics showcased in Table 4, significant differences appeared in the planning activities (*P* = 0.047*, 0.011* <0.05) including sharing success criteria with students before classroom instruction. More specifically, younger university teachers (assistants and lecturers) enacted the strongest professional agency in planning, and associate professors exercised the lowest agency either in planning or practicing, which were far smaller than younger teachers.

### Result of qualitative research data

#### Exertion of professional agency in planning

In the first round of the research cycle, English department teachers with different experiences in teaching enacted different levels of professional agency in planning when they took decisions, made actions, and responded to the existing teaching reforms, especially when comparing preservice teachers with those teaching experts. The wide disparity contributed to the discussion of the exertion of agency in planning, and reasons were reported based on their justifications of different reactions, related to iterational and practical-evaluative dimensions. Teachers’ previous knowledge and basic ideas on the teaching practices, and references to the curriculum plan resources could be the major effects in the planning section.

##### Sharing criteria with learners

In the light of ecological interpretation of professional agency, the material dimension refers to resources (timetable, classroom, and teaching resources, among others) and physical environment, which is well worth further considering. Teachers expressed difficulty in designing the school-related and students’ development-oriented resources based on the higher level of teaching resources. With considerable autonomy in the university class, teachers would challenge to take actions and decide how to activate student’s motivation in learning, especially in some elective courses. Teachers who enacted a weak sense of agency argued that the practices of the curriculum-designed class could consume teachers’ attempts when contextualizing the materials to develop students’ capacity in planning.

*I teach one of elective courses for English majors called “western films,” cultivating students’ appreciation of good films and beauty in the actors’ story. It can benefit students’ sense of beauty and justice. However, some students could not sense the significance of lesson, treated it as a time-killer. Hence, while planning, I need much time and thinking in lesson plans, considering how to conduct the lesson attractively and value-provokingly. …Existing resource can certainly encourage me to design proper plans along with the whole semester. However, how to design and conduct an effective lesson still burdens me heavily (IN 6, June 2021).*

Meanwhile, teachers enable to enjoy the privilege of access to the school support and colleague relationship in the process of planning. The structure and influence of the relations between colleagues or administrators could either bolster or deter the professional agency, which depends on the development of teacher identity in the learning community. Preservice teachers in universities face more challenges in training themselves to be active agents in developing their professional identities as they lack enough teaching practices. When asked about their proactive actions in the practicum, one teacher commented that:

*It is often that teaching feedback could not match the previous planning, which could make me doubt myself. Luckily, my school and colleagues could have supports (both substance and emotion) available to go through the dark days. Since I recognize the role and responsibility of being a university teacher, the enactment of professional agency could be enhanced to develop the quality of teaching by the collaborative and positive relationships (IN 3, June 2021).*

When asked about their purposes in the planning, teachers have various beliefs but all are concerned with learning to be professional educators or subjective teaching experts.

*When in the pre-servicing, I pushed myself to be both professional educators and teaching expert, and I could both develop students’ academic ability by help construct their knowledge and enhance my capacity as a professional educator. That would company with my whole teaching career. However, when I design my teaching planning, the main focus would lay down on the learning and teaching, how to construct students’ knowledge and what methods could I possibly apply in my classroom within the different learning contexts (IN 1, March 2021).*

From teachers’ concepts of teaching purposes in the planning, teachers might exercise the long-term purposes in cultivating students’ competence and teaching them how to learn, which significantly showcases the relatedness of developing novice teachers’ identity. Considering the unstable nature of teacher identity, teachers’ professional development highly depends on how teachers enact their agentic capacity in dealing with variables at different levels.

*Novice teachers need to adapt themselves to the university teaching life quickly, and to develop their professional capacity in the limited time (normally 2 or 3 years). Hence, in the first few years, little time would be spent on the teaching practices. Thanks to the assistance and interaction within learning community of university, the problem could be alleviated (IN 7, March 2021).*

On account of teachers’ justification for decision-making in planning, the discourse analysis seems to bind the iterational dimension (beliefs and ways of working) with a practical-evaluative statement of agency (relationship). This accountability of agentic exertion suggested that teacher enhanced their capacity in the context of the learning community rather than in an isolated situation. In order to better understand the nature of this learning community in which teachers enact their agency to promote teachers’ professional development, teachers in the first research cycle would be interviewed again during a teaching in the midterm and after assessment activities in the final term.

#### Exertion of professional agency during teaching

##### Classroom questioning

After the classroom observation, teachers seemed to adopt the questioning activity as the mediator to facilitate students’ participation, aiming to arouse their attention, interests, and motivation when they were drawn to outside of the classroom.

*Young students would be easily lost in the prevalence of Internet, and it is common to find students devoting themselves in the cellphone, chatting with others, shopping…hence, the application of questioning in the classroom might make them tense, nervous, and participated. Besides, it could also bind the relationship between students and teachers as it involves kind of interaction in the classroom and develops the quality of lesson (IN 2, April 2021).*

Teachers’ interpretation of classroom questioning announced that teacher would enact their professional agency in the classroom questioning. Based on the further discussion, should classrooms have the availability of structural and material factors such as psychical resources and their supplements, and collaborative interrelatedness while teaching, teachers would enact strong levels of professional agency by means of participating in the classroom, which struck a note of practical significance in teaching.

##### Comment-only marking

Subsequent events along with the questioning in the classroom call for timely feedback, which could either be positive or negative and albeit impact notably on learning and achievement. Fortunately, university teachers (in the humanities and social science branches) have acknowledged the value of comment types of marking. As teachers commented on the difference between humanities and social science, and science, some significantly obvious words like multi-dimension, multi-perspective, and open questions could be retrieved from the discourse analysis.

*Although it is bite non-science to tell the difference between humanities and science, as they all belong to human historical experience. However, with the development of society and economy, the need to solve the tricky questions using the more professional knowledge stands out and this knowledge gap requires immediate resolution. To my knowledge, subjectively albeit, the aim or purpose of humanities and social science is to cultivate individual’s self-understanding and enhance their development by encouraging them to express their concepts on the previous knowledge. So, I would not advise other teaches to use one answer to the question. It’s not one plus one, but how to understand concept on your own (IN 10, April 2021).*

Teachers who have built their teacher identity empower to comment students’ achievements without mere scores while teaching and mark their development with the methods of providing suggestions based on teachers’ understanding. There is only one difference between teachers and students, elder teachers have a splendid future behind them, and the young students have a glorious future before them. Hence, that determines that teachers’ goal is to bridge the gap between them. Interestingly, when asked about their agency in the marking, the majority of teachers show the same attitudes, suggesting that teachers would enact a strong sense of agency in the comment-only marking.

#### Exertion of professional agency in assessment

##### Peer-and self-assessment

Teachers in the interviewing seemed to showcase that although they have comprehended the necessity of self-cognition and self-assessment, the whole assessment activities lack enough time-related contribution.

*University teachers have way too much power to decide students’ trajectory and evaluate whether they have reached the designed level, which is beneficial for learning autonomy albeit, might neglect students’ agentic actions on decision-making. To my opinion, the power of self and peer assessment is out of our imagination. Once students are able to know what the different between better students and their current stage, their motivation would be activated in order to reach the sense of identity and they could be accepted in their community. Hence, why some teachers not let students try to enact their peer and self-assessment in the classroom from time to time (IN 9, June 2021).*

The peer-and self-assessment involves the practical-evaluative dimensions of variables in the enactment of professional agency, related to the interactions among students in the collaborative context. Furthermore, some suggestions could be highlighted from teachers’ discourse (bilateralness), that is, the formation of formative assessment activities ought to not only exert teachers’ leading role in class (maximally excavating teachers’ competence) but also take students’ agentic role into consideration (making students’ feel at ease in class).

##### Formative use of summative tests

The impact of student-based formative assessment practices on the performance of university English major students in summative tests has been evaluated and emphasized in the learning potential of assessment. However, the long history of exam-oriented context in China makes it much more challenging to implement the assessment for learning purposes. Thanks to the university’s context, teachers would have more power to adopt the formative assessment strategy for enhancing students’ learning and treat it as an effective method of teaching and learning.

*In the text-dominated culture, summative assessment is believed to undermine the design and purpose of formative assessment. Teachers have toiled in the practices for building a reliable and effective synergy between formative and summative assessment. And we would apply the summative test performance as adequately as possible, for example, we would design summative tests for the purpose of producing formative information et al. (IN 5, June 2021).*

Other teachers also expressed their passion for the design of summative tests in accordance with the relatedness of formative use of summative performance. They employed summative tests to identify students’ learning difficulties and assist students’ further development in capacity, which suggests that they would exercise a strong level of professional agency in the formative use of summative performance in the final term of teaching.

## Discussion

### Teachers’ experience of professional agency in formative assessment

**FIGURE 3:**
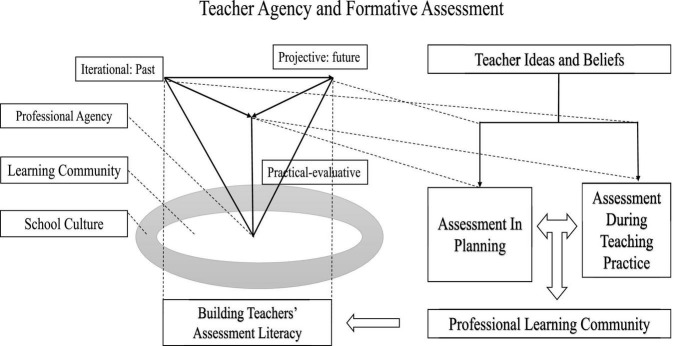
Teachers’ enactment of professional agency in the implementation of formative assessment activities.

In light of the two systems of professional agency and formative assessment, some hypotheses about interrelatedness are be assembled in Figure 3.

In alignment with current research, teachers in the study found value in formative assessment activities to develop students’ learning and enhance their own teaching capacity, which emphasized the promotion and facilitation of professional agency for sustainability in the teaching context.

When implementing the planning activities, teachers experience the collaborative culture in the community as materializing future-related orientations with reference to the development-focused curriculum, prior life history, and culture of learning. In this period, teachers enact their professional agency on account of futural directions, where collaboration and communication have been stressed for the realization of shared professional strengths and peer mentorship. Following the projective privilege in the futural orientations, teachers’ personal perceptions and practices could be amended over time for professional development only if under the right support, administrative leadership, and peer collaboration, which involves the prevalence of teachers’ past experiences. Teachers’ sense of agency could be greatly promoted by the synergy between past performance and current compensation.

University school life has continued to accentuate the interactive collaboration in “nature kindergarten” as a place of learning community where layers of stakeholders, such as student learners, faculty, and administrative leaders, grow and learn together with a unified mission. Sharing experience and professional development opportunities has been perceived as valuable in the professional learning community; such sharing includes both preservice educational training and collaborative discussion among faculty members on which assessment literacy could be depended and built on.

### Barriers and facilitators to enacting teachers’ professional agency

The university community provides teachers with great opportunities and challenges, but young teachers struggle with various sources of pressure, including workload, physical and mental health, leadership, career development, and scientific research pressure. The investigation of the nature of university English teachers’ experiences and post-activities bridges the gap in the current situation of scientific research on university teachers. This empirical study of teachers’ agency both spatially and temporally depends on the university context in which teachers live and work. Hence, the ecological framework of teacher agency enhances agentic involvement because each teacher is supported or restricted to some extent by the social environment, material availability, and emotional impact of the surroundings (Priestley et al., 2015). Demographic characteristics of the target university teachers in quantitative research, as well as teachers’ comments on their interpretation of teacher agency and formative assessment activities, are jointly presented to assist administrators and researchers to bridge the gap in understanding potential enablers and restraints to university English teachers’ agency.

Apart from three significant themes in the process of teaching, each section has three specific domains based on the ecological-driven categorizations that still have statistically different and symbolic features. In fact, teachers enact the highest level of past experience and professional competency (iterational variable) compared with the projective and practical-evaluative variables, in which teachers enact a sense of agency in terms of purposes and teachers’ evaluation of the physical or cultural environment. To put it more straightforwardly, in the case of planning of teaching and learning activities and agency in the formative use of summative tests, teachers report a statistically similar pattern of how the agency is distributed in different domains: projective agency prior to practical-evaluative domain but minor to iterational one. Notwithstanding, in the process of practicing classroom activities (in which teachers guide learners on how to participate and get involved in the classroom and ask for feedback to adjust the teaching process and time allocation), teachers support the importance of the physical environment and resources together with cultural and structural enactment (practical-evaluative domain).

**FIGURE 4:**
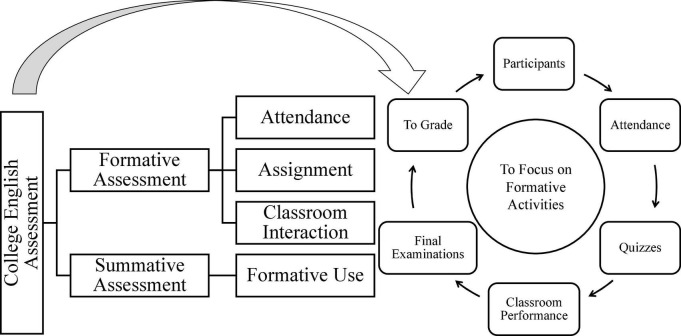
University formative assessment framework.

Some intriguing results could be outlined from various perspectives in relation to university English teachers’ agency. Formative assessment and summative assessment in the final tests are the two interplaying factors when evaluating students’ improvement in one learning journey. Teachers enacted the highest sense of agency in the process of assessment because of the method of interpreting formative assessment in universities. In response to university education reform and assessment initiatives, the target university formed and implemented a new evaluation mechanism (Figure 4) during the teaching process. In contrast with traditional summative assessment, classroom interaction, attendance, performance in class, and quizzes became the important factors to be included and assessed in the final curriculum evaluation. School administrators and teachers have been informed of and have acknowledged the significance of process assessment, which has made them realize that teachers are active motivators and facilitators who could shape learners’ characters during a long learning period.

This description and interpretation of teacher agency in different phases of teaching practices gave an overview of the essence of how teachers’ agency was distributed and enacted while participating in agentic activities. Further factors that enabled or constrained the realization of teachers’ professional agency are discussed in the next subsection with the aim of spotting and identifying the deeper layer of practical variables in relation to teachers’ mental activities.

#### The effects of demographic characteristics on university English teachers’ professional agency

Currently, efforts are being made to unveil the relationship between demographic features and determine whether teachers’ professional agency varies considerably in accordance with demographic variables. In this study, demographic data were analyzed using descriptive analysis and displayed as frequencies to demonstrate the relatedness of seniority, gender, age, educational qualifications, and professional title.

##### Variable one: The relation between teacher seniority and agency

Broadly, no matter in which sub-domains, teachers who have long teaching experience enacted lower professional agency compared with younger teachers since the average mean scores descend sharply or marginally in an analogous way. On average, university teachers would have more effective power and agentic involvement over the first years of their teaching career at the university (Rivkin et al., 2005; Boyd et al., 2011). Normally, teachers improve to a large extent over the first several years due to the need to produce satisfying results and avoid scientific-research-driven layoffs. Therefore, the inexperienced and younger teachers would put more effort into self-development with full vitality. However, the evaluation mechanism in Chinese universities easily results in job burnout after years of experience and a lack of necessary agency in teaching activities, for too much focus had been maintained on the number of published papers (paper-centric evaluation of professional titles in university). To narrow this evaluation gap, the Chinese government has implemented reforms to provide more opportunities for effectiveness in teaching and feature more varieties to activate and retain teachers’ agency.

Still, the analysis uncovered that there was a significant imparity in the agency in the planning domain. The agency in planning teaching activities decreased sharply during the fresh years of teaching careers but increased slightly when their competence and capacity reached a certain extent. Further inquiry into sub-dimensions revealed that iterational and projective dimensions covered the most percentage of significant difference, but not in the practical-evaluation dimension. From the statistical outcomes of agency in formative assessment, younger teachers have the agentic tendency to show a higher level of professional experience and teaching goals (either short-term or long-term) in the teaching practices. Nonetheless, the other two processes demonstrate no close correlation in relation to a professional agency.

##### Variable two: The impact of gender on teacher agency

Drawing on a statistical analysis of mean score and SD data, female teachers enacted a higher level of professional agency in the three phases of formative assessment activities compared to male teachers. In the process of formative use of summative tests, both female and male teachers got similar scores, which indicated that teachers have more passion to engage in evaluating students’ performance, for it could provide direct and positive feedback to teachers’ teaching capacity. However, it is certainly worth noting that teaching procedure is a motive and dynamic interaction in which more attention needs to be given to the planning and particularly teaching performance. In other words, gender as the mediating role in the enactment of professional agency in the language teaching practices shapes future aspirations along with other contextual and situational variables (Vitanova, 2018).

To put it specifically, in many circumstances female teachers confront various capacity constraints than male teachers (Tao, 2019), in which social norms appoint women as the ones who should assume the responsibility for children rearing and domestic chores. Luckily, female teachers have certainly enacted agency to surmount and reclaim opportunity for achievement and development, which results in higher agency in the projective domain in educational contexts than male teachers. Furthermore, since there are gender gaps and constraints in working, the policy and educational practice targeting encouraging female teachers to challenge the social bias and stemming the negative effects of constraints should be implemented to access more opportunities for women teachers (Practical-Evaluative Dimension).

##### Variable three: The impact of age on teacher agency

The demographic variable of age was exploited with the method of ANOVA analysis to argue whether university teachers of different ages would enact the distinctive agency in the formative assessment. After statistical data processing and examination of the results, it revealed that teachers experienced different levels of agency in the three phases of teaching activities, albeit with a vague statistical linear distribution. Overall, the detailed and comprehensive display bore a little resemblance to the effects of years of teaching experience.

Teachers, especially those during the “teacher educational or pre-service educational year,” have witnessed a growing educational development in the construction of university teacher teams, with the purposes of increasing teacher development or efficacy beliefs (Hoy and Spero, 2005) and decreasing the professional burnout (Van Dick and Wagner, 2001), which could extensively promote the academic development of pupils. To this end, they augmented a higher sense of agency to pledge the desirable existence of promotional opportunities, pay-rises, and the excitement of being involved in a new and dynamic environment (workplace).

Additionally, the preceding research conducted by Akif (2016) utilized a similar confirmatory factor analysis to reveal the relatedness of the demographical variables with work engagement in Turkey and observed that there was no significant importance between work engagement and age, gender, and educational qualifications. Instead, the impact of seniority had been stressed and enjoyed the significant importance of work engagement, which means that teachers with more teaching experience would have more agency to engage with the activities, but the variables of age represent no agentic action to current situations.

##### Variable four: The effect of professional title on teacher agency

In the demographic realm of variables in the enactment of professional agency, the professional title is another factor that could facilitate or prevent teaching and learning with the method of activating or deactivating the agentic mechanism while taking action or making reactions in regard to changes. The research of professional title (determinism or influents) characterizes differences among a variety of teachers with different levels of professional titles shown as mean scores in the qualitative study. Normally, in the regions of Chinese university teachers’ title evaluating system, a developmental teacher evaluating system aiming to boost teachers’ professional development has been introduced and founded on professional attitude, awareness, knowledge, and capacity (Chen and Xu, 2006). These four aspects contribute to the acquisition of professional titles which were connected with diverse degrees of job satisfaction. Hence, a great deal of attention has been called to teachers’ professional development (title) concerning job pay, promotional opportunities, and job security (Ramos et al., 2021).

Regarding professional titles with different professional experiences in teaching, the results demonstrated significant differences, which suggested co-relations between teacher agency and the variable of professional titles that existed in the planning activities. To present in another approach, comparably, teachers with lower titles (lecturer or associate professor) had higher agency than professors, but this might not be adequately evidential for the imbalance in participants. Teachers with lower titles could be probably confronted with more chances of layoff and research pressures, which led to lower job satisfaction. To settle this matter, they would exercise higher levels of the agency to bear the thought of professional development and achievement in academic output. Apart from the disparity of professional experience, formative use of summative tests gained the most agentic focus in the teaching process, and the other two domains had lower agency within the mean scores. It would be sensible to notify professors who tended to have more agency and focus on the teaching assessment phase.

#### Synopsis of variables in formative assessment activities

To summarize the influence over the exercise of teacher agency in the process of formative assessment, a collection of variables was reviewed and spelled out for the joint effects on the demography-driven judgment and reaction to the teaching environment. Most strikingly, teachers of different gender enjoyed different levels of agency in the phase of formative use of summative tests at the end of the semester, and female teachers would have more tendency to activate themselves for teachers’ professional development and students’ academic advancement. As for teachers with richer teaching experience, higher levels of agency could be enacted to render the planning of teaching activities effective and fruitful, and there was a decreasing tendency along with the teaching practice. Moreover, there was statistics-driven evidence of links between age-associated factors and enactment of professional agency, which identified that novice teachers would exercise much more agency to attain the extent to which they could sense job satisfaction with the growth of teaching experience and prevent dropout throughout training and retention of staff.

University English teachers could exercise a strong sense of professional agency in classroom instruction, and some variables have been targeted and explored for the impact of teachers’ proactive choices toward ecological conceptualization. The previous discussion of variance in the enactment of the professional agency has raised concerns about the discoursing effects on the development of teachers’ competency in teaching and facilitating students’ knowledge construction.

Enough classroom observation and individual interviews suggested that the enablers and constraints in conducting formative assessment had coalesced into the real situation of classroom instruction when teachers enacted professional agency. Teachers with more support from collegiate leaders and in a collaborative environment could exercise strong professional agency, which means that teachers who enjoyed a free teaching atmosphere might have more motivation in the classroom. Furthermore, should university teachers have more time in classroom interaction with students and conduct face-to-face teaching activities, teachers might have more tendency to enact their agency. However, the need to prioritize the differentiated and flexible management of time stands out to affect the enactment of professional agency. It is acknowledged that teachers demand a flexible and adjustable time allocation mechanism in the course of planning and teaching activities, along with collegiate collaboration. Moreover, based on their experience, teachers are suggested that time is required to be devoted to teaching reflection on the teaching pedagogies and strategies.

When considering how to evaluate students’ achievement after attending the lecture-based instrument or flipped learning, university teachers spontaneously consider the accountability and efficacy of formative assessment for the fairness of student assessment practices. University teachers appear to concentrate more on students’ performance instead of summative tests scores in final exams. Analysis of teachers’ experiences in formative assessment activities revealed that they normally utilize their professional agency with short-term aspirations as a projective dimension to materialize and form the collegiate-led, teacher-dominant formative assessments. The usage of formative assessment companied with summative assessment could largely liberate teachers’ choices in planning and teaching, which means that formative assessment could contribute to a stronger sense of professional agency. Another finding in the formative assessment is teachers’ assessment literacy. The occurrence of assessment literacy in teachers’ discourse analysis brings to the problem of how to build teachers’ assessment literacy for the sake of high-quality assessments. In response to teachers’ more professional agency in enhancing students’ achievement, some actions should be performed based on their experience. For example, professional learning, problem-centralized instructions, and assessment-led practices should function accordingly.

## Conclusion and implications

Considering the constraints and enablers in the enactment of professional agency in formative assessment, demographically speaking, novice teachers might exercise comparatively stronger agency for retention and teacher development, with an irregular downward trajectory in the implementation of formative assessment activities. In particular, teachers’ aspirations to strengthen their assessment literacy and assessment capacity (projective dimension of agency) could heavily promote reflection and decision making in the formative assessment. In the context of university teaching and planning, teachers are encouraged to use summative tests as a formative assessment because summative tasks challenge professional experiences and teachers’ beliefs (the iterational and the practical-evaluative). Moreover, teachers’ professional agency could be enabled by differentiated and flexible allocation in the teaching experience, especially in planning for participating in teaching reflection and collaboration. Were limited time contributed to the reflection and formative assessment activities, it would be difficult to materialize teachers’ sense of assessment literacy or promote teacher development with enough teaching exercises and feedback. Collaborative, positive, and informal relationships in the classroom and collegial contexts that are built on trust, accountability, and honesty could be another influential enabler of exercising teachers’ professional agency, highlighting the impact of the practical-evaluative dimension.

In response to participants’ experiences of professional agency, the point in time and contexts determine a unique situation in which material, cultural, and individual factors bind together to guide the trajectory of decision making. The analysis of planning experiences accentuated the interactive influence of projective (either short or long term) and practical-evaluative dimensions of professional agency in the implementation of formative assessment activities. In individual classroom practices, teachers’ responses and actions depend on iterational and practical-evaluative dimensions of professional agency. Teachers in the experiment reported that in combination with the re-evocation of past life experiences and knowledge, authentic collegial relationships and strong support (practical-evaluative) enabled them to judge and make decisions about how to react and prioritize in pedagogical practice. Along with the planning and teaching sections, teachers’ reflection and decision making in the assessment-related activities, known as formative assessment, would highlight the effects of strong professional agency in the learning community to foster teachers’ assessment literacy, which is conclusively founded on teachers’ development.

Teachers’ development, which has been promoted by the enactment of strong professional agency, could be implicated in teaching and learning practices. As teachers identify, respond to, and reflect on their teaching and learning experiences, their sense of identity might have a mediating effect, while teachers come to be active agents in developing professional identities. In response to changes in direction and decision making in the university teaching context, teachers would reach back into their pasts to identify key moments that had contributed to where they found themselves at present and guide them to the future (Tedder and Biesta, 2007; Ruohotie-Lyhty and Moate, 2016).

Under the guidance of experienced teachers, novice teachers can experience the stage of cooperative teaching in which their learning capacity and teaching skills are enhanced and developed. If the implementation of formative assessment could greatly support teachers’ professional decision making (as there is no need to be confined by the summative assessment standards), the enactment of professional agency in the English teaching context would be the major factor in developing professional learning. Hence, professional-learning-oriented activities should consider possible opportunities for reflective and collaborative discussion before, during, and after teaching, and it should highlight the major contributors and benefits that could help build positive working relationships.

Despite the constraints of structural, material, and cultural variables in the university context, teachers are involved in the enactment of stronger experiences of professional agency in planning and teaching practices. Accordingly, teachers ought to decide on reflection to adjust their pedagogical strategies in formative assessment activities. Ultimately, teachers should consider and seize on available opportunities to materialize projective prospects in decision making along with planning, exerting, and assessing. In this study, teachers in the planning and teaching period experienced little collegial cooperation and enjoyed the privilege of controlling learning progression, which lacks essential feedback in accordance with assessment activities. It is suggested that university teachers should reflect on the assessment actions and proceed with collaborative construction in the learning community. Should teachers concentrate on effective feedback and adjustment in pedagogical strategies and methods, their sense of assessment literacy and professional development might be promoted to a greater extent. In addition, collaborative interaction requires teachers’ agentic participation. Teachers are expected to become involved in cooperative communication within a positive working environment to help expand the range of innovative experiences and insights. Hence, teachers need to contemplate how to construct a positive collaborative learning community bound with the goal of achieving professional development. Lectures, keynote speeches, and other academic seminars could provide opportunities to foster positive relationships between faculty colleagues, and teachers should pay much more attention to the supplementary impacts of these academic activities. In turn, positive relationships in academia can activate and maintain strong levels of professional agency in teachers’ teaching practices and academic reflections.

School administration has played an indispensable and vital role in the enactment of professional agency because material resources could constrain decision making. Some alternatives to administrative decisions should be available if teachers experience a sense of constraint, which suggests that school leaders ought to provide a collaborative work environment and enhance teachers’ trust in the context of a collegial relationship with the university. This supportive context can facilitate the enactment of teachers’ professional agency with enhanced self-efficacy and lessened anxiety within increasingly friendly relations (Gilbert et al., 2014; Liu et al., 2021). Since teachers’ professional agency relies greatly on social interrelations and interactions between peers, which materialize the exertion of reflections and professional learning, collaborative contexts could help promote the enactment of professional agency in a learning community. Hence, school leaders should take the initiative in constructing and strengthening positive relationships in the community where teachers can share their trust and develop their learning capacity. Under teaching development, schools and collegial institutions should provide necessary guidance to teachers, helping them adjust to the learning community in the university. Such assistance may include financial support for academic exchanges and seminars in the same loop. Teachers in this study also reported that limited temporal contribution to reflections and collaborations may stifle the exercise of agency. As such, teachers should be given enough time for planning, learning, and reflecting on their practice according to the modifications and adjustments in the school years.

## Considerations for further study

With regard to further study, it is suggested that the relatedness of identity and agency is worth further consideration to present the whole picture of teacher development. Arguably identity is often identified as an abstraction or a freeze-frame of the history-based constellation of self within a particular point of time while the agency is related to the incessant negotiation with identity-oriented activities (Vähäsantanen, 2015; Ruohotie-Lyhty and Moate, 2016). The constant negotiation and realization of teacher identity within the serving experiences in university makes the notion of professional agency coherent and understandable in promoting teachers’ development in the learning community (Li and Jiang, 2021). The conceptualization of teacher identity could also benefit the ongoing research on why teachers’ assessment literacy in the formative assessment could strengthen the exercise of a professional agency.

## Data availability statement

The raw data supporting the conclusions of this article will be made available by the authors, without undue reservation.

## Ethics statement

Ethical review and approval was not required for the study on human participants in accordance with the local legislation and institutional requirements. Written informed consent from the patients/ participants or patients/participants legal guardian/next of kin was not required to participate in this study in accordance with the national legislation and the institutional requirements.

## Author contributions

All authors listed have made a substantial, direct, and intellectual contribution to the work, and approved it for publication.
