# Supplementary material for: An ecological approach to understanding university English teachers’ professional agency in implementing formative assessment
Source: Front Psychol. 2022 Sep 14;13:916980. doi: 10.3389/fpsyg.2022.916980 (PMC9521544; doi:10.3389/fpsyg.2022.916980)
Supplement: Supplementary file 1 [file Data_Sheet_1.docx]

**Supplementary Material**

Supplementary Appendix 1: Questionnaire of University English Teachers’ Professional Agency

**Basic information including:** *Gender, Age****,*** *Teaching Years****,*** *Educational Background****,*** *Professional Title.*

**University English teachers are asked to rate to what extent they agree with the following statements on 5-point Likert scale (1 - strongly disagree; 5 - strongly agree)**

**Part One Planning Teaching and Learning Activities**

*Iterational dimension:*

(1) I find based on my studies that planning teaching and learning activities is valuable.

(2) I’m experienced in planning teaching and learning activities.

(3) I feel confident in planning teaching and learning activities.

*Projective dimension:*

(4) I have clear long-term purposes for planning teaching and learning activities.

(5) I have clear short-term purposes for planning teaching and learning activities.

(6) Planning teaching and learning activities is for me a personally meaningful aim (I feel that this is valuable).

*Practical-evaluative dimension:*

(7) Organizational culture (e.g., competition or collaboration oriented) at my (practice) school supports planning teaching and learning activities.

(8) I experience good administration support (e.g., timetable, workload, size of class) for planning teaching and learning activities at my (practice) school.

(9) I experience good support from colleagues for planning teaching and learning activities at my (practice) school.

(10) My (practice) school has good material resources (e.g., computers and other equipment) for planning teaching and learning activities.

**Part Two Implementing Teaching and Learning Activities**

*Iterational dimension:*

(1) I find based on my studies that implementing teaching and learning activities is valuable.

(2) I’m experienced in implementing teaching and learning activities.

(3) I feel confident in implementing teaching and learning activities.

*Projective dimension:*

(4) I have clear long-term purposes for implementing teaching and learning activities.

(5) I have clear short-term purposes for implementing teaching and learning activities.

(6) Implementing teaching and learning activities is for me a personally meaningful aim (I feel that this is valuable).

*Practical-evaluative dimension:*

(7) Organizational culture (e.g., competition or collaboration oriented) at my (practice) school supports implementing teaching and learning activities.

(8) I experience good administration support (e.g., timetable, workload, size of class) for implementing teaching and learning activities at my (practice) school.

(9) I experience good support from colleagues for implementing teaching and learning activities at my (practice) school.

(10) My (practice) school has good material resources (e.g., computers and other equipment) for implementing teaching and learning activities.

**Part Three Using Formative Assessment in Teaching and Learning Activities**

*Iterational dimension:*

(1) I find based on my studies that using formative assessment in teaching and learning activities is valuable.

(2) I’m experienced in using formative assessment in teaching and learning activities.

(3) I feel confident in using formative assessment in teaching and learning activities.

*Projective dimension:*

(4) I have clear long-term purposes for using formative assessment in teaching and learning activities.

(5) I have clear short-term purposes for using formative assessment in teaching and learning activities.

(6) Using formative assessment in teaching and learning activities is for me a personally meaningful aim (I feel that this is valuable).

*Practical-evaluative dimension:*

(7) Organizational culture (e.g., competition or collaboration oriented) at my (practice) school supports using formative assessment in teaching and learning activities.

(8) I experience good administration support (e.g., timetable, workload, size of class) for using formative assessment in teaching and learning activities at my (practice) school.

(9) I experience good support from colleagues for using formative assessment in teaching and learning activities at my (practice) school.

(10) My (practice) school has good material resources (e.g., computers and other equipment) for using formative assessment in teaching and learning activities.

*Questionnaire is adapted from Leijen, Ä. et al. 2021 (Leijen, Ä., et al. Assessing Student Teachers’ Agency and Using it for Predicting Commitment to Teaching [J]. European Journal of Teacher Education, 2021: 1-17.)*

**Supplementary Appendix 2: Interview Protocol of University English Teachers’ Professional Agency**

*Purpose of the interview: To gain further insights into the essential contents and interconnectedness of teacher agency*

*Method of interview: Semi-structured*

*Interviewees: Some of English teachers who participated in the questionnaire*

**Part One: Basic Information**

Which school did you graduate from? What was your major? How does your major fit in with your current career?

What do you think of teaching English in higher education?

How do you position yourself as a college English teacher?

Is your school able to meet the requirements for English career development? Does your school provide certain avenues for further education and development?

**Part Two: The Situation where University Teachers Enact their Professional Agency in Formative Assessment**

Are you familiar with formative assessment? How formative assessment activities are carried out in classroom?

What problems have you encountered in conducting formative assessment activities? what solution have you applied to these problems?

When you encountered problems, what you were thinking?

**Part Three: Factor which Influence the Enactment of Professional Agency**

What factors might affect your professional agency as a teacher? What are the reasons for this?

Do school environment and learning support affect your daily teaching?

Does your school conduct regular professional learning activities for teachers? Do you think it has any effect on your daily teaching?

**Part Four: Individuals’ Understanding of Professional Agency**

To what extent is your current teaching style influenced by your previous learning experiences?

To what extent is your current teaching style influenced by your expectations of future teaching?

To what extent is your current teaching style influenced by the surrounding teaching environment and teaching facilities?

What factors have influenced you in your personal development as a teacher?
